# Stakeholder Perspectives on Telehealth Effectiveness, Facilitators, and Barriers for Wheelchair Service Provision: A Needs Analysis

**DOI:** 10.63144/ijt.2025.6682

**Published:** 2025-06-12

**Authors:** Rachel Taylor, Richard Schein, Corey Morrow

**Affiliations:** 1 Department of Occupational Therapy, East Carolina University, Greenville, North Carolina, USA; 2 Department of Rehabilitation Science and Technology, University of Pittsburgh, Pittsburgh, Pennsylvania, USA; 3 Division of Occupational Therapy, Department of Rehabilitation Sciences, Medical University of South Carolina, Charleston, South Carolina, USA

**Keywords:** Mobility, Telehealth, Telerehabilitation, Wheelchair services

## Abstract

Mobility impairments limit access to wheelchair services, especially in rural settings. Telehealth offers one potential solution to improve access. The purpose of this paper is to describe the current perceptions of telehealth wheelchair follow-up services among a select group working in the industry. A 35-question survey was completed by 32 stakeholders in the wheelchair industry to describe their perceptions of telehealth effectiveness, facilitators, and barriers for wheelchair provision and follow-up. Telehealth was generally perceived as effective for reaching rural populations, but specific subpopulations and technology requirements should also be considered. Respondents also indicated which Common Procedural Terminology (CPT) billing codes are commonly used for wheelchair services. The results of this survey will be used to design a quality improvement project within a rural healthcare system. This project will aim to improve access to wheelchair follow-up services via telehealth that are feasible and sustainable for the local healthcare system.

Mobility impairments affect approximately 40 million U.S. adults (Centers for Disease Control and Prevention, 2023). Without adequate intervention (Wee & Lysaght, 2009), these mobility impairments are associated with negative health outcomes including falls (Musich et al., 2018), a reduction in health-related quality of life (Davis et al., 2015), and decreased access to specialized care (Lagu et al., 2013). The negative impacts of mobility impairments may disproportionately affect rural populations that have to travel longer distances to receive specialized care. Specialized medical providers are often unavailable in rural areas, so access requires significant travel (Mullens et al., 2023), potentially resulting in healthcare access inequities (Hahn et al., 2023; Morrow et al., 2024; Murimwa et al., 2023; Williams et al., 2016).

To address mobility impairments, physical therapy and occupational therapy are often consulted to implement remediation interventions (i.e., strengthening and balance retraining). When mobility impairments cannot be remediated because of factors such as injury severity or a progressive disease process, healthcare professionals may recommend compensatory mobility devices such as canes, walkers, and standard wheelchairs. Additionally, persons with severe mobility impairments may benefit from specialized wheelchairs to facilitate participation in daily life (Rousseau-Harrison & Rochette, 2013).

Specialized wheelchair service provision is a complex process that ideally involves “referral, assessment, equipment recommendation and selection, funding and procurement, product preparation, fitting, training and delivery, follow-up maintenance and repair, and outcome measurement” (RESNA, 2011). Wheelchair service provision also should involve multiple stakeholders including users, therapists, suppliers, Assistive Technology Professionals (ATPs), and payors (Betz et al., 2022; Schein et al., 2021). The Rehabilitation Engineering & Assistive Technology Society of North America (RESNA) provides best-practice recommendations for appropriate wheelchair service provision to match custom-fit wheelchairs with patients’ functional status (RESNA, 2011). However, barriers to the implementation of best-practice wheelchair service provision include long wait times, specialist shortages, and limited consumer education (Betz et al., 2022). Wheelchair users not only experience these issues during the initial evaluation process but also when they require follow-up and repairs to these complex devices (Henderson et al., 2022; Ruffing et al., 2024). Across the four- to five-year lifespan of the wheelchair, wheelchair users seek follow-up repair services 5.10 times for manual wheelchairs and 8.42 times for power wheelchairs (James et al., 2023). Active follow-ups from a clinician have been shown to significantly reduce the rate of incidents related to the need for repair (Hansen et al., 2004). However, clinicians specialized in wheelchair service provision may be inaccessible to rural populations.

Telehealth services may be one option to improve access for wheelchair users (Ruffing et al., 2024) living in rural specialty shortage areas and overcome barriers to the implementation of follow-up services such as travel burden (Hahn et al., 2023). Telehealth uses telecommunication technologies to improve healthcare access and delivery (Winters, 2002), including virtual visits, chat-based interactions, remote monitoring, and technology-enabled modalities (American Telemedicine Association, 2020). Studies conducted with Veterans have demonstrated that telehealth for wheelchair service provision has been beneficial for reducing travel burden (Ott et al., 2022) without a negative effect on clinical outcomes (Bell et al., 2020). While these studies have shown the benefits of telehealth services, the Veteran Affairs (VA) healthcare system only covers 1.0% of the U.S. population (Keisler-Starkey et al., 2023). The VA also has a different funding structure and serves different patient populations than the Centers for Medicare and Medicaid Services (CMS), which covers a majority of U.S. citizens with a disability (U.S. Census Bureau & U.S. Department of Commerce, 2023). The effectiveness of telehealth services for wheelchair service provision among the populations served by CMS has not yet been established in the literature. During the COVID-19 pandemic, CMS issued emergency authorization for telehealth services, enabling further study of telehealth usage for wheelchair service provision (McClammer et al., 2024).

Despite the potential benefits, barriers to the implementation of equitable wheelchair follow-up may hinder adoption. Potential barriers to telehealth include reimbursement, staff support, technology requirements, and technology literacy of both clients and their caregivers (Cortelyou-Ward et al., 2020; Lyu et al., 2022). Reimbursement is of particular concern for many healthcare organizations (Shachar et al., 2020) to maintain financial viability. Although CMS issued emergency authorization during the COVID-19 pandemic, concerns may persist for telehealth services as reimbursement has been historically variable across payers (Dahl-Popolizio et al., 2020). For wheelchair follow-up services, identifying commonly used and reimbursable billing codes for wheelchair services may help standardize reimbursement and mitigate this barrier. Additionally, data from the COVID-19 pandemic suggests disparities in access to video-based telehealth services (Lee et al., 2023), potentially due to a lack of reliable broadband access in rural areas (Federal Communications Commission, 2020). More information is needed regarding barriers to telehealth wheelchair services post-pandemic. For rural populations in professional shortage areas, further exploration is needed on the feasibility of telehealth as an alternative to in-person wheelchair follow-up.

The purpose of this project was to identify stakeholder perspectives surrounding a telehealth approach to wheelchair service provision and guide the development of a quality improvement project in a rural healthcare system. A needs assessment via an online survey was completed by stakeholders in the wheelchair industry to understand their perceptions of telehealth effectiveness, facilitators, and barriers in adult wheelchair service provision including follow-up.

## Methods

This needs assessment was part of a quality improvement (QI) project at a large academic medical center. A 35-item online survey was designed and managed using the Research Electronic Data Capture (REDCap) software hosted at Medical University of South Carolina (Harris et al., 2009, 2019) for secure data collection.

### Question Development

Questions were drafted by one clinician and one clinician-researcher with expertise in the field of wheelchair service research. A second clinician and another clinician-researcher with expertise in the field of wheelchair service research reviewed the survey for content and face validity (Gasque et al., 2024). They provided feedback on the survey’s usability, including word choice, use of a 4-point Likert scale, and survey flow.

The survey collected demographic information about the respondents’ profession(s), current role(s), primary population served, type of clinic where they currently work, and previous experience with telehealth for wheelchair evaluation and follow-up. Demographics were collected by multiple-choice and multi-select questions with a free-text box provided for qualitative clarification when “Other” was selected. Since healthcare professionals often serve multiple roles, such as clinician and researcher, and can hold multiple credentials simultaneously, such as occupational therapist and Assistive Technology Professional, demographic questions about profession and roles were designed to be multi-select.

Quantitative and qualitative questions examined concepts of perceived telehealth effectiveness, facilitators, and barriers for telehealth wheelchair provision (*See* Appendix). Quantitative data included multiple-choice, 4-point Likert scale (Brown, 2010), and multi-select questions. When respondents indicated “Other” or “N/A,” a free-text box was provided for qualitative clarification.

### Recruitment and Inclusion Criteria

Potential respondents were recruited both locally and nationally using a snowball sampling methodology (Ghaljaie et al., 2017). Locally, respondents were recruited via word-of-mouth. Since this was part of a quality improvement project, one clinician, one manager, and two suppliers working within a rural healthcare system in rural North Carolina were recruited for participation. Nationally, an email was sent to 20 clinicians who are subject matter experts in seating and mobility, with a request to forward to known researchers, clinicians, managers, and suppliers in the field. The online survey was open from July 2, 2024, to July 16, 2024.

Regardless of whether they had provided telehealth services, respondents were included in the final aggregate data if they had completed or otherwise been involved with patient care for wheelchair services. Respondents were excluded if they had never been involved with wheelchair provision or if they were students.

### Data and Ethics

No identifying data or protected health information was collected from participants, and respondents were reminded that participation was voluntary. Aggregated data is presented to ensure that responses are anonymous. This project was deemed a quality improvement project by the Medical University of South Carolina Institutional Review Board’s QI/Program Evaluation Self-Certification Tool.[Fig f1-ijt-17-1-6682]

## Results

A total of 32 individuals completed this survey and met the inclusion criteria. Due to the snowball sampling method, the response rate cannot be determined. Respondents (N = 32) were primarily occupational therapists (n = 12, 37.5%) or physical therapists (n = 16, 50.0%) who completed wheelchair evaluations, and one-third of respondents were certified as an Assistive Technology Professional (n = 11, 34.4%). The most common role was clinician (n = 28, 87.5%). Respondents served a variety of populations, including urban (n = 9, 28.1%), suburban (n = 14, 43.8%), and rural (n = 9, 28.1%) communities (*See* Table 1).

### Perceived Effectiveness

Among respondents who indicated that they had used telehealth for wheelchair service provision in the past (N = 24), 16.7% (n = 4) perceived that telehealth was effective for wheelchair evaluations every time they have used it, 70.8% (n = 17) perceived that telehealth was effective only in the right situation, and 12.5% (n = 3) perceived that telehealth was not effective.

At least 80% of those who had used telehealth for wheelchair services agreed or strongly agreed that telehealth was effective for the following situations: reaching underserved populations, rural populations, and older adults; providing information on supplemental funding sources for parts or equipment not covered by health insurance; and measuring outcomes after a wheelchair evaluation/device delivery. Contrastingly, at least 50% of respondents disagreed or strongly disagreed that telehealth was effective for reaching adults with acutely acquired mobility impairments and providing transfer training.

### Facilitators/Barriers

**Figure 2:**
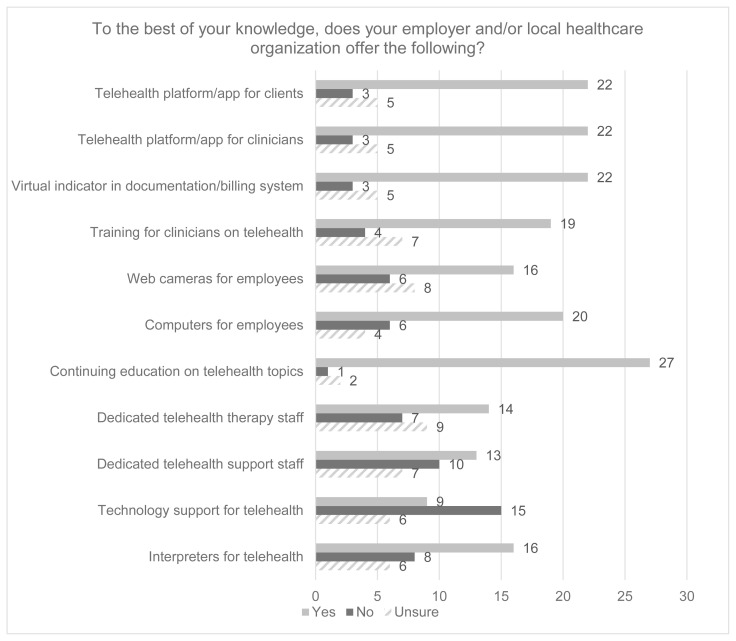
Facilitators of General Telehealth Services

Two respondents did not respond to the Facilitators/Barriers section of the survey. The remaining respondents (N = 30) were asked to identify which of the potential facilitators listed were available at their facility, regardless of whether they offered telehealth services. At least 50% of respondents indicated that basic hardware (i.e., computers, webcams), software (platform, apps, EHR integration), and training were available to the clinicians at their facility. However, 33.3% of respondents indicated that their facility did not have support staff specifically trained or focused on telehealth service delivery, and 23.3% indicated their facility did not have therapy staff specifically trained or focused on telehealth service delivery (*See* Figure 2).

**Figure 3:**
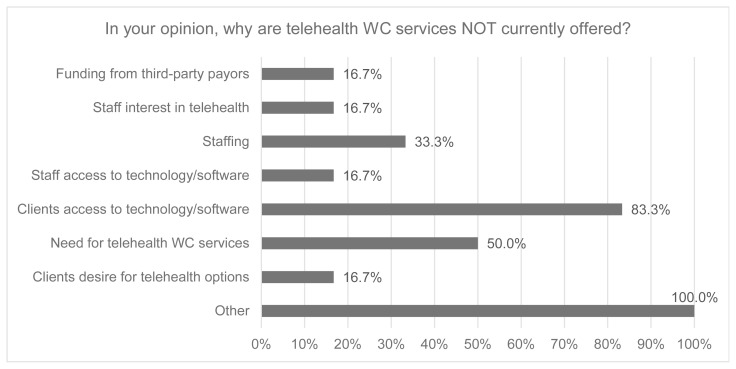
Barriers to Telehealth Use for Wheelchair Evaluations Note. WC = wheelchair

Respondents who indicated that their healthcare organization did not offer telehealth for wheelchair evaluation and follow-up (N = 6) were asked to identify perceived barriers to providing telehealth services (*See* Figure 3). The most substantial barrier identified was that clients do not have access to the appropriate technology/software (83.3%). For respondents who indicated “Other” barriers, qualitative data further identified credentialling requirements of the facility/payors, clinician perceptions of effectiveness, and the novelty of telehealth for clinicians completing wheelchair evaluations as barriers to telehealth for wheelchair provision.

### Reimbursement

Respondents who had completed wheelchair evaluations or follow-up sessions in the past were asked which Current Procedural Terminology (CPT) codes are used for billing telehealth or in-person sessions. The most common codes used for evaluation were CPT codes 97542: Wheelchair management (54.8%); 97165-7 for Occupational therapy evaluations (41.9%); and 97161-3 for Physical therapy evaluations (45.2%). Other codes used included CPT codes 97755: Assistive technology assessment; 97530: Therapeutic activities; 97110: Therapeutic exercise; 97112: Neuromuscular reeducation; and 97535: Self-care. Of note, one respondent indicated that using CPT code 97542: Wheelchair management alongside an evaluation code has not been covered in the past.

The most common code used for follow-up services was CPT Code 97542: Wheelchair management (73.3.%). Other codes included 97755: Assistive technology assessment, 97530: Therapeutic activities, 97535: Self-care, and 97750: Physical performance test. CPT codes 97168 for occupational therapy re-evaluations and 97164 for physical therapy re-evaluations were also used when the session occurred outside the plan of care or certification period. Of note, one respondent indicated that billing CPT code 97542: Wheelchair management resulted in a zero-dollar payment for their center. No other mention of reimbursement rate was made.

## Discussion

This project surveyed professionals working in the wheelchair industry to determine the current needs and perceptions surrounding telehealth wheelchair services. The results can be used to guide future directions for both research and quality improvement for rural health systems.

### Perceptions of Effectiveness

Telehealth, in general, has the potential to reduce healthcare costs or increase revenue by allowing clinicians to see a higher volume of patients, reducing avoidable hospitalizations, and reducing wait times for those in rural areas (Lillicrap et al., 2021). While telehealth may not always be appropriate, the benefits of telehealth for wheelchair provision should be explored as a standard option in rural healthcare systems and as a reimbursable service for payors.

A majority of the stakeholders perceived that telehealth is an effective method overall for reaching a rural population for wheelchair evaluations and follow-up services. This perceived effectiveness is supported in the literature. A scoping review of telehealth delivery of remote assessment of wheelchair and seating needs concluded that preliminary research suggests telehealth wheelchair assessment may be as effective as in-person assessment and is viewed favorably by wheelchair users and nonspecialist assessors (Graham et al., 2020). Earlier studies suggest that wheelchair service delivery via telehealth may be as effective as in-person evaluations in reaching decisions about wheelchair and seating modifications and prescriptions (Malagodi et al., 1998; Schein et al., 2010). Despite this, there are still other practical aspects to consider. Within rural communities, there were variations in perceived effectiveness among subpopulations, including acute traumatic mobility impairments, congenital mobility impairments, and first-time wheelchair users. Though telehealth may increase access to rural areas, clinical reasoning should still be used when deciding the appropriateness of providing wheelchair services via telehealth.

Beyond access, telehealth may be perceived as effective for a variety of reasons. Telehealth with video functions offers the outpatient clinician an opportunity to see inside the wheelchair user’s home environment. However, a majority of respondents perceived that transfer training would not be effective to complete via telehealth. This finding may conflict with literature on patient-reported outcomes. One study found that patient satisfaction with transfers in the home were higher among Veterans who received a custom wheelchair via telehealth as compared to an in-person group of Veterans (Bell et al., 2020). Telehealth may be particularly beneficial for transfer training in the wheelchair user’s home environment. Further research is needed to determine the relationship between telehealth and transfer training, including safety considerations, appropriate patient population, best practice, and potential telehealth advantages.

### Facilitators/Barriers Identified

Facilitators and barriers of telehealth may also have impacted stakeholder perceptions of telehealth effectiveness. Though clinicians perceived their clinics to have adequate technology – including webcams, computers, and telehealth platforms – they perceived client access to technology as the most significant barrier to telehealth services. Further research may be needed to differentiate between client access to technology and clients’ technology literacy, which was not assessed in this survey. Dedicated clinicians and support staff for telehealth were not common, but not all respondents worked at clinics where telehealth was currently offered. In a traditional outpatient rehabilitation setting, lower-cost support staff are responsible for administrative tasks. This allows therapists to spend more time providing billable services to patients which may lead to lower overall service delivery costs (Morrow et al., 2023). Additional research is needed to determine the impact of staffing ratios and staff training on telehealth wheelchair service implementation and perceived effectiveness.

However, the perceived lack of client technology access highlights the need for client-centered interventions rather than health system-based interventions. These client-centered interventions may include offering both video and audio-only telehealth options, providing training on available technology, or providing essential technology for telehealth sessions. The American Telemedicine Association’s Principles for Delivering Telerehabilitation Services (Richmond et al., 2017) published a best practice document that highlights many of the same facilitators and barriers previously described. Specifically, this best practice document was to inform and assist practitioners in providing effective and secure services that are based on client needs, current empirical evidence, and available technologies. In addition, this document serves as a resource for developing discipline-specific standards, guidelines, and practice requirements.

### Reimbursement Considerations

Among those surveyed, there was a wide variety of CPT codes used and reimbursement received for wheelchair services, whether in-person or telehealth. With such wide variability in reimbursement and the need for clinicians to hold advanced certification for wheelchair provision (Schein et al., 2021), it may be difficult for health systems to initiate and sustain specialized wheelchair services. Correct coding and documentation for therapy reimbursement are critical to the success of any outpatient clinic or home health provider. Qualitative results of this survey demonstrate a perceived fear of zero-dollar payment or denial when using CPT Code 97542: Wheelchair management. Large data research is needed on the consistency of reimbursement rates between rural and urban/suburban health systems, how CPT codes are being utilized for wheelchair provision, and what factors of the healthcare systems or policy may impact these variations.

While many health insurance companies follow CMS guidelines (Centers for Medicare & Medicaid Services, 2023), variations exist among modifiers used and reimbursement rates for telehealth services. Data from this online survey can provide a starting place for determining billing codes that could be used for comparable telehealth services, with the addition of appropriate modifiers. It is best practice to check with your professional association and payors to make sure which CPT codes and telehealth modifier to use prior to billing (American Occupational Therapy Association, 2018).

### Future Directions

The results of this survey will be used to design a quality improvement project within a local rural health system. Additional investigation of the responses will influence the accessibility of wheelchair follow-up services for local wheelchair users, as well as the feasibility and sustainability of a telehealth approach for the health system.

### Limitations

Several limitations deserve discussion. First, the recruitment sampling was a type of nonrandom approach that relied on the research team’s network size. Participants in this study were limited to the organization’s outreach from the original emails. The results represent the opinions of a select group of stakeholders, not a representative sample. Second, the survey was conducted as part of a quality improvement project and therefore results are not generalizable to the larger wheelchair service industry or rural health community.

## Conclusion

This project sets the direction for quality improvement within the rural healthcare system studied and highlights the need for research on the feasibility of telehealth wheelchair services in rural healthcare systems. With travel burdens disproportionately affecting rural populations, research is needed to understand the feasibility of providing specialized wheelchair services via telehealth when in-person services are inaccessible. Additionally, the current survey highlights the need for further research to explore which aspects of wheelchair provision are most appropriate for telehealth.

## Figures and Tables

**Figure 1 f1-ijt-17-1-6682:**
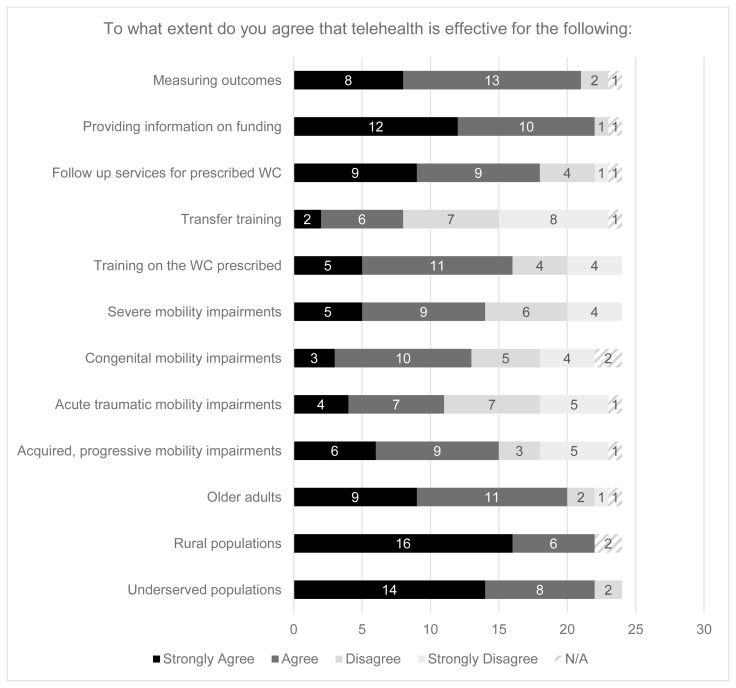
Stakeholder Perceptions of Telehealth Effectiveness *Note.* WC = wheelchair; respondents were asked about the effectiveness of providing these services or reaching these populations

**Table 1:** Demographics of Respondents

	n	%
Profession(s)		

Occupational therapy	12	37.5%
Physical therapy	16	50.0%
Assistive Technology Professional	11	34.4%
Engineer	1	3.1%
Other^1^	1	3.1%

Role(s)		

Clinician	26	81.3%
Management	9	28.1%
Academia/Research	6	18.8%
Supplier/Sales	2	6.3%

Primary Population		

Rural	9	28.1%
Suburban	14	43.8%
Urban	9	28.1%

Type of Clinic		

Private practice outpatient	5	15.6%
Large academic medical center	17	53.1%
Large non-academic medical center	2	6.3%
Other^2^	8	25.0%

Telehealth Experience		

Yes, but not for WC evaluation and/or follow up	1	3.1%
Yes, and I have used it for WC evaluation and/or follow up	24	75.0%
No, I have never used it	7	21.9%

*Note*. WC = wheelchair;

1 Seating and Mobility Specialist;

2 “Other” included inpatient rehabilitation facilities, schools, group homes, the Veteran’s Administration, pro-bono clinics, and outpatient clinics associated with other types of facilities and universities.
